# Association Between Overweight Sarcopenic Population and Acute Vertebral Osteoporotic Compression Fractures in Females: Retrospective, Cross-Sectional Study

**DOI:** 10.3389/fmed.2021.790135

**Published:** 2021-12-03

**Authors:** Younghun Lee, Ho-Jae Lee, Siyeong Yoon, Jaeyeon Shin, Kyung-Chae Park, So-young Lee, Soonchul Lee

**Affiliations:** ^1^Department of Orthopaedic Surgery, CHA Bundang Medical Center, CHA University School of Medicine, Seongnam, South Korea; ^2^Department of Orthopaedic Surgery, CHA Gumi Medical Center, Gumi, South Korea; ^3^Department of Computer Science, College of IT Engineering, SeMyung University, Jecheon, South Korea; ^4^Department of Family Medicine, Health Promotion Center, CHA Bundang Medical Center, CHA University School of Medicine, Pocheon, South Korea; ^5^Department of Internal Medicine, CHA Bundang Medical Center, CHA University School of Medicine, Seongnam, South Korea

**Keywords:** sarcopenic obesity, osteoporosis, vertebral compression fracture, bone mineral density, body composition test

## Abstract

**Background:** This study aimed to determine whether the prevalence of acute vertebral osteoporotic compression fractures (VOCF) in the elderly population is related to the distribution of muscles and fat in the human body.

**Methods:** Data of acute VOCF and non-VOCF patients presenting at our institution between January 2018 and May 2020 were analyzed. Patients aged 65 years and older, who underwent body composition test and dual-energy X-ray absorptiometry at the same time were enrolled. After applying exclusion criteria, patients were divided into four groups: normal, sarcopenia without obesity, obesity without sarcopenia, and sarcopenic obesity. Body mass index ≥25 kg/m^2^ was considered obesity, and sarcopenia was defined as skeletal muscle index lower than 7.0 kg/m^2^ in males and 5.4 kg/m^2^ in females. The VOCF rate was analyzed between the groups.

**Discussion:** A total of 461 patients were included, of whom 103 were males. Among them, 163 (35.36%) had normal body composition, 151 (32.75%) had sarcopenia without obesity, 110 (23.86%) had obesity without sarcopenia, and 37 (8.03%) had sarcopenic obesity. The sarcopenic obesity group had the highest rate of acute VOCF (37.8%), which was statistically significant. Specifically, females with sarcopenic obesity and sarcopenia without obesity had significantly higher acute VOCF rates compared to those with normal body compositions. Multivariate analysis showed that sarcopenic obesity was significantly associated with acute VOCF rate overall, as well as in females.

**Conclusion:** Sarcopenic obesity is strongly associated with acute VOCF, especially in females, and it could be an essential criterion for the prevention of acute VOCF.

## Introduction

Vertebral osteoporotic compression fracture (VOCF) causes a reduction in vertebral body height and is most frequently seen in the thoracolumbar transition zone (1). Symptomatic fractures often lead to severe pain, deformity, decreased mobility, decreased pulmonary function, and increased risk of age-adjusted mortality. Therefore, evidence-based prevention and management are essential (2). However, with the increase in the senior population, VOCF has also been increasing. In the United States, 1.5 million fractures are attributed to osteoporosis every year; more than 700,000 Americans are annually diagnosed with vertebral fractures (3, 4). It is estimated that at least 25% of American women reaching menopause experience at least one VOCF in their lifetime (5).

Many risk factors associated with VOCF have been reported. Risk factors are categorized into not modifiable, such as age, gender, and race, and potentially modifiable factors such as alcohol use, osteoporosis, early menopause, and malnutrition (6). Recently, sarcopenia has been frequently studied as a risk factor for VOCF. Sarcopenia is a syndrome characterized by reduced muscle volume, muscle strength, and muscle activity. It has begun to attract attention in various medical fields. In addition to the vertebral compression fractures, multiple factors and diseases have been studied in relation to sarcopenia (7–9). Additionally, sarcopenia has been shown to be significantly associated with osteoporosis, and the decrease in muscle content, strength, and function substantially increased the risk of osteoporosis (10–12).

Sarcopenic obesity is a combination of two words: sarcopenia and obesity. Appendicular lean mass (see Diagnosis Criteria for Sarcopenia and Obesity section) is used to define sarcopenia. If sarcopenia coexists with excess body fat, it is called sarcopenic obesity (13). Numerous reports have been published regarding this syndrome, and several studies have evaluated its relationship with other diseases such as cardiovascular disease and solid tumors (14, 15). Furthermore, many disorders affecting metabolism, physical capacity, and quality of life have been linked to sarcopenic obesity, though whether sarcopenia and obesity act synergistically has yet to be determined (16). In particular, there are no previous studies on the relationship between sarcopenic obesity and VOCF.

In this cross-sectional study, we collected the data of patients who underwent dual-energy X-ray absorptiometry (DEXA), including body composition tests, and analyzed the rate of acute VOCF according to the groups divided by criteria of bone mineral density (BMD) and muscle volume. With the difference in the prevalence of acute VOCF and adequate statistical methods, we aimed to determine the relationship between body composition category and acute VOCF.

## Methods

### Study Design and Patients

**Figure 1:**
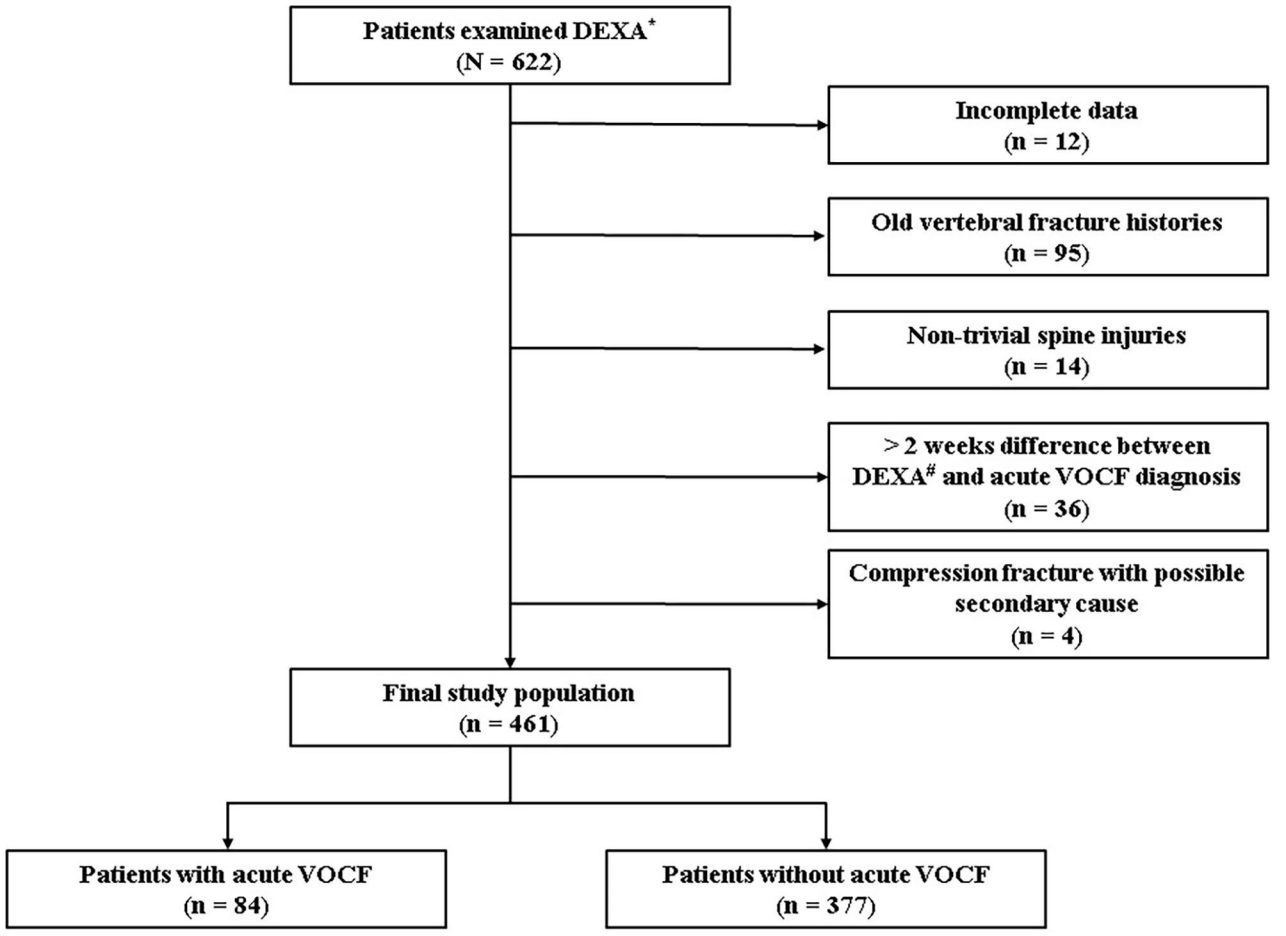
Flowchart. *Dual energy X-ray absorptiometry, ^#^vertebral osteoporotic compression fracture.

After institutional review board approval (No. 2021-02-019), we initially set up the range of the reference group. Informed consent for participants is waived. We included patients who were presented to the spine center and health promotion center at the CHA Bundang Medical Hospital between January 2018 and May 2020. For the patients who visited the spine center, those who were diagnosed with acute VOCF, based on history taking, physical examination, and spine radiography, was included in the experimental group (Acute VOCF group). We only assumed that the patient had the acute VOCF when the patient had the recent trauma history and the tenderness on the fracture site without callus formation on the spine radiography. Finally, we created a list of patients aged 65 years and over, who underwent DEXA with body composition tests at the same time. On the other hand, patients who visited the health promotion center were selected as a reference group. Since people over 65 can receive insurance coverage in our country. These patients took the same tests but did not have acute VOCF on spine radiography. As it was designed as a retrospective study, a total of 622 patients were enrolled initially. After detailed history taking and baseline evaluation, the patients were excluded based on the following exclusion criteria: 1. Patients with incomplete data (*n* = 12); 2. Patients with old VOCF history (*n* = 95) since we only focused on acute VOCF; 3. Patients with high-energy injury mechanisms, such as severe traffic accidents or falling from a tall height (*n* = 14) as most patients presented with mild back pain or other soft tissue injuries; 4. Patients who underwent body composition tests more than 2 weeks from the diagnosis of acute VOCF (*n* = 36) since the treatment of acute VOCF can influence the patient's nutrition and activity so that the body mass index and body composition can change; 5. Patients with possible secondary cause, such as bone metastasis and primary hyperparathyroidism (*n* = 4). Finally, a total of 461 patients were included in the statistical analyses (Figure 1).

### Diagnosis Criteria for Sarcopenia and Obesity

Patients and healthy participants were divided into four groups: normal, sarcopenia without obesity, obesity without sarcopenia, and sarcopenic obesity. The primary anthropometric data, height and weight, were obtained. Body mass index (BMI) was calculated as body weight divided by squared height, and it was used as an index of obesity. Participants with BMI ≥ 25 kg/m^2^ were considered obese according to the Korean cut-off values, since it is defined as standard guideline in Korea, related to clinical outcome (17, 18). Next, the body composition test results were analyzed to define and categorize the sarcopenic group. The sum of the muscle masses of the four limbs was defined as appendicular skeletal muscle mass (ASM), and skeletal muscle index (SMI) was defined as ASM/height (m)^2^. To measure ASM, the results of 4 limbs from body composition test were used and [Sum of lean mass] (g) – [Sum of bone mineral content] (g) is the exact calculation method. Among various cut-off values from numerous studies, and especially for ASIA consensus, SMI lower than 7.0 kg/m^2^ in males and 5.4 kg/m^2^ in females were employed (19).

### Variables

Age, gender, BMI, height and weight, body composition, and T-score at the lumbar vertebrae and hip were obtained. T-scores were measured using DEXA (GE Lunar Prodigy, Madison, WI, USA) and were calculated by taking the difference between a patient's measured BMD and the mean BMD in healthy young adults, matched for gender and ethnic group, and expressing the difference relative to the standard deviation (SD) of the young adult population (20). In addition, DEXA has become a method of choice for assessing body composition because of its unique ability to conveniently and efficiently measure distinct body compartments, including total and regional fat mass, lean tissue mass, bone mineral content, etc. (21). In addition, with the electronic medical record browsing, each participant's history, such as hypertension, diabetes mellitus, and cancer, was assessed. Laboratory results were also collected; reference levels and units are as follows. The detailed values of serum calcium (8.6–10.2 mg/dL), phosphorus (2.5–4.5 mg/dL), vitamin D (30–100 ng/ml), and parathyroid hormone (PTH) (15–65 pg/ml) were obtained. All samples are from the venous blood and each result is from specialized detection devices. Serum calcium and phosphorus levels were measured by Stat Profile® Critical Care Xpress analyzer (Nova Biomedical, Waltham, MA, USA). A 25(OH)D_3_-specific kit (Cobra II Auto-γ Counting System, Packard Instruments, Downers Grove, IL, USA) was used for vitamin D measurement. Also, we measured Serum PTH levels by standard enzyme-linked immunosorbent assay-PTH immunoradiometric assay (IBL International GmbH, Hamburg, Germany) with 1.0 pg/ml as a minimal detection amount.

### Statistical Analyses

The data manipulation and statistical analyses were performed using R software (version 3.6.3; The R Foundation for Statistical Computing, Vienna, Austria; http://www.R-project.org/). Continuous, normally distributed data are presented as mean and SD. Statistical significance was set at *p* < 0.05. We used the unpaired t-test and Pearson's chi-squared test to compare characteristics of subjects according to the gender. We also used Pearson's chi-squared test to confirm the difference between distributions of the body composition by gender. Univariate analysis was performed to determine the factors associated with acute VOCF. In addition, we performed *post hoc* analysis using Fisher's exact test, which is appropriate for handling small sample sizes, and compared the four body composition groups according to the presence or absence of acute VOCF. Multivariate logistic regression analysis was used, adjusting for factors and covariates to confirm the simultaneous effect of multiple factors associated with acute VOCF in female. Normal body composition was used as the reference category. Results were presented as odds ratios (ORs) with 95% confidence intervals (CIs) and Akaike Information Criteria (AIC) values, which estimate the quality of each model.

## Results

### Demographic Characteristics and Body Composition Categories Between Genders

Among 461 patients, 103 were males and 358 were females. Anthropometric factors such as height, weight, and ASM were significantly different between males and females. In addition, significant differences were observed in the T-scores for both the spine and hip between male and female groups. The females had the T-scores of −2.0 ± 1.2 in the spine and −1.9 ± 1.1 in the femur, and both were significantly lower compared to the males that are −1.1 ± 1.5 in the spine and −0.9 ± 1.4 in the femur (*p* < 0.001) (Table 1).

**Table 1:** Demographic characteristics between both genders.

**Variable**	**Male (*n* = 103)**	**Female (*n* = 358)**	***p***
**Age**	70.6 ± 8.6	70.5 ± 9.7	0.969^*^
Height (m)	1.66 ± 0.61	1.54 ± 0.06	<0.001^*^
Weight (kg)	63.9 ± 10.5	56.5 ± 9.5	<0.001^*^
BMI (kg/m^2^)	23.1 ± 3.5	23.8 ± 3.6	0.102^*^
Exam date—Diagnostic date (days)	39.3 ± 90.0	30.7 ± 153.6	0.776^*^
ASM (kg)	19.5 ± 3.1	13.9 ± 2.3	<0.001^*^
SMI (kg/m^2^)	7.1 ± 1.1	5.9 ± 0.8	<0.001^*^
**T-score**			
Spine	−1.1 ± 1.5	−2.0 ± 1.2	<0.001^*^
Hip	−0.9 ± 1.4	−1.9 ± 1.1	<0.001^*^
**Lab**			
Calcium (mg/dl)	9.0 ± 0.6	9.1 ± 0.6	0.145^*^
Phosphorus (mg/dl)	3.2 ± 0.6	3.5 ± 0.6	<0.001^*^
Vitamin D (ng/ml)	19.3 ± 8.9	23.8 ± 11.6	0.004^*^
PTH (pg/ml)	40.5 ± 19.5	45.6 ± 23.0	0.527^*^
**Comorbidity**			
Hypertension			0.574^**^
Yes	48 (46.6%)	153 (42.9%)	
No	55 (46.6%)	204 (57.1%)	
Dyslipidemia			0.108^**^
Yes	12 (11.7%)	68 (19.1%)	
No	91 (88.3%)	288 (80.9%)	
Diabetes mellitus			0.564^**^
Yes	22 (21.4%)	65 (18.2%)	
No	81 (78.6%)	292 (81.8%)	
Cancer			0.565^**^
Yes	8 (7.8%)	20 (5.6%)	
No	95 (92.2%)	337 (94.4%)	

* *T-test*,

** *Pearson's chi-squared test*.

Regarding body composition category, ~30–40% of the study population was in the normal body composition group, irrespective of gender. The proportion of subjects with obesity, with or without sarcopenia, was higher in the female group. In contrast, the rate of sarcopenia, with or without obesity, was higher in the male group. Consequently, among the total patients, 163 (35.36%) had a normal body composition, 151 (32.75%) had sarcopenia without obesity, 110 (23.86%) had obesity without sarcopenia, and 37 (8.03%) had sarcopenic obesity with *p* < 0.001 by Pearson's chi-squared test. The details of the proportions of subjects in each of the four body composition groups are summarized in Table 2.

**Table 2:** Distribution of the four body composition categories by gender.

	**Normal** ***n*** **(%)**	**Sarcopenia** **without obesity** ***n*** **(%)**	**Obesity without** **sarcopenia** ***n*** **(%)**	**Sarcopenic obesity** ***n*** **(%)**	**Total**	***p*** ^*^
**Gender**						0.037
Male	32 (31.07)	42 (40.78)	17 (16.50)	12 (11.65)	103 (100.0)	
Female	131 (36.59)	109 (30.45)	93 (25.98)	25 (6.98)	358 (100.0)	
Total	163 (35.36)	151 (32.75)	110 (23.86)	37 (8.03)	461 (100.0)	

* *Pearson's chi-squared test*.

### Differences Between Acute VOCF and the Healthy Controls

Among the 461 patients, 84 had acute VOCF and 377 did not. The average age was significantly higher in the acute VOCF group than in the healthy control group (74.7 vs. 69.6 years old, *p* < 0.001). The T-scores of the acute VOCF group were significantly lower than those of the healthy control group (*p* = 0.002 for the spine and *p* < 0.001 for the hip). In addition, calcium and phosphorus were also significantly different between the groups (8.7 vs. 9.2 mg/dL, *p* < 0.001 for calcium and 3.2 vs. 3.5 mg/dL, *p* < 0.022 for phosphorus). However, co-morbidities, such as hypertension and diabetes mellitus, were not significantly different between the two groups. In the normal group, 14.1% of the patients had acute VOCF. The sarcopenia without obesity group had a fracture rate of 22.5%. The obesity without sarcopenia group had the lowest fracture rate (11.8%). Finally, the sarcopenic obesity group had the highest fracture rate (37.8%). Pearson's chi-squared test demonstrated that the fracture rate was significantly different among these four groups (*p* = 0.003) (Table 3).

**Table 3:** Univariate analysis of factors associated with VOCF.

**Variable**	**Acute VOCF** (***N*** **= 84)**	**Non-VOCF** (***N*** **= 377)**	***p***
**Age**	74.7 ± 9.9	69.6 ± 9.1	<0.001^*^
**BMI (kg/m^**2**^)**	23.4 ± 3.6	23.6 ± 3.6	0.648^*^
**Body composition category**			<0.001^**^
Normal	23 (14.1%)	140 (85.9%)	
Sarcopenia without obesity	34 (22.5%)	117 (77.5%)	
Obesity without sarcopenia	13 (11.8%)	97 (88.2%)	
Sarcopenic obesity	14 (37.8%)	23 (62.2%)	
**T-score**			
Spine	−2.2 ± 1.3	−1.7 ± 1.3	0.002^*^
Hip	−2.2 ± 1.3	−1.6 ± 1.2	<0.001^*^
**Lab**			
Calcium (mg/dl)	8.7 ± 0.9	9.2 ± 0.5	<0.001^*^
Phosphorus (mg/dl)	3.2 ± 0.7	3.5 ± 0.6	0.022^*^
Vitamin D (ng/ml)	19.5 ± 11.1	23.4 ± 11.1	0.050^*^
PTH (pg/ml)	38.5 ± 15.4	48.7 ± 25.4	0.077^*^
**Comorbidity**			
Hypertension			0.301^**^
Yes	41 (49.4%)	160 (42.4%)	
No	42 (50.6%)	217 (57.6%)	
Dyslipidemia			0.757^**^
Yes	13 (15.7%)	67 (17.8%)	
No	70 (84.3%)	309 (82.2%)	
Diabetes mellitus			0.386^**^
Yes	19 (22.9%)	68 (18.0%)	
No	64 (77.1%)	309 (82.0%)	
Cancer			0.080^**^
Yes	9 (10.8%)	19 (5.0%)	
No	74 (89.2%)	358 (95.0%)	

* *T-test*,

** *Pearson's chi-square test*.

Next, a *post-hoc* analysis with Fisher's exact test was applied to assess fracture rate of each body composition group compared to that of the normal body composition group. In the entire study population, a significant difference was found only between normal and sarcopenic obesity (*p* = 0.002). Regarding the females, we observed that both sarcopenia without obesity and sarcopenic obesity groups had significantly higher rates of acute VOCF than the normal group (*p* = 0.018 and *p* = 0.001, respectively). However, in the male group, there were no statistically significant correlations in the *post hoc* analyses (Table 4).

**Table 4:** *Post hoc* analysis of the four groups by VOCF.

	**Acute VOCF** ***n*** **(%)**	**Non-VOCF** ***n*** **(%)**	***p*** ^*^		
**Total**	84 (18.2%)	377 (81.8%)			
Normal	23 (14.1%)	140 (85.9%)	Reference		
Sarcopenia without obesity	34 (22.5%)	117 (77.5%)	0.058	Reference	
Obesity without sarcopenia	13 (11.8%)	97 (88.2%)	0.716	0.033	Reference
Sarcopenic obesity	14 (37.8%)	23 (62.2%)	0.002	0.062	0.001
**Male**	15 (14.6%)	88 (85.4%)			
Normal	4 (12.5%)	28 (87.5%)	Reference		
Sarcopenia without obesity	6 (14.3%)	36 (85.7%)	1	Reference	
Obesity without sarcopenia	2 (11.8%)	15 (88.2%)	1	0.532	Reference
Sarcopenic obesity	3 (25.0%)	9 (75.0%)	0.391	0.735	0.193
**Female**	69 (19.3%)	289 (80.7%)			
Normal	19 (14.5%)	112 (85.5%)	Reference		
Sarcopenia without obesity	28 (25.7%)	81 (74.3%)	0.018	Reference	
Obesity without sarcopenia	11 (11.8%)	82 (88.2%)	0.703	0.042	Reference
Sarcopenic obesity	11 (44.0%)	14 (56.0%)	0.001	0.045	0.001

* *Fisher's exact test*.

### Multivariate Logistic Regression Analysis of Factors Associated With Acute VOCF

**Figure 2:**
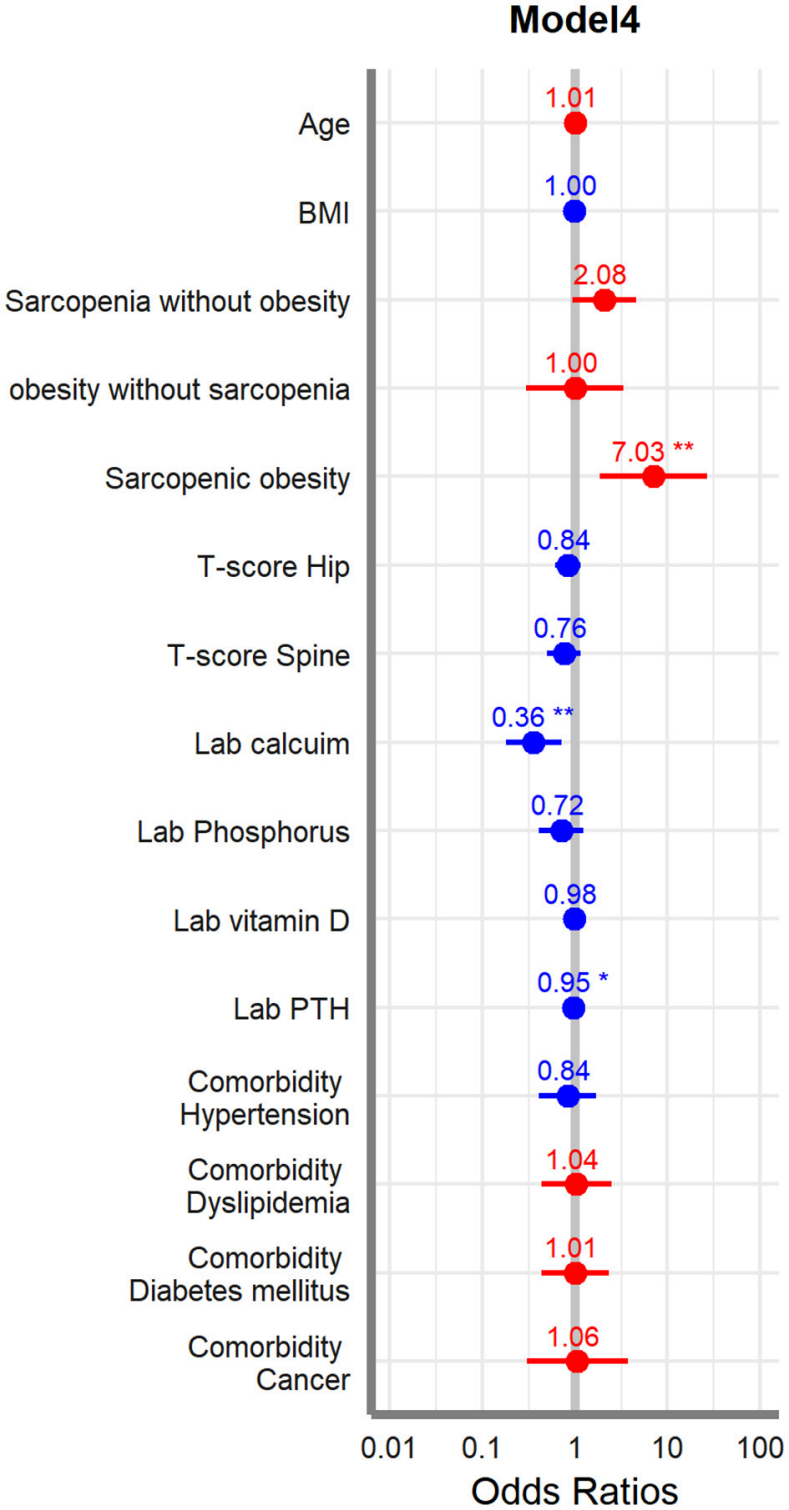
Forest plot of multivariate regression analysis (female).

Finally, we used multivariate logistic regression adjusting for factors and covariates such as age, BMI, four different body composition categories, T-score, laboratory results, and comorbidities. Models are divided into 4 ways, and Model 1 has only basic variables of the patients; other variables are gradually added to the other models and have roles as confounding factors so Model 4 has all variables included. In the *post hoc* analysis result, only female patients showed that sarcopenic obesity group has significant result compared to any other subgroups; we did this multivariate regression for the females. The results showed that the sarcopenic obesity, calcium, and PTH were significantly associated with acute VOCF (*p* < 0.05). Sarcopenic obesity was especially significant in all the models (Table 5). Forest plot was drawn for indicating the odds ratio of each variable (Figure 2).

**Table 5:** Multivariate logistic regression analysis of factors associated with acute VOCF for female.

**Variable**	**Model 1**		**Model 2**		**Model 3**		**Model 4**	
	**AIC** **=** **291.86**		**AIC** **=** **288.13**		**AIC** **=** **275.28**		**AIC** **=** **283.03**	
	**OR (95% CI)**	***p***	**OR (95% CI)**	***p***	**OR (95% CI)**	***p***	**OR (95% CI)**	***p***
**Diagnosed age**	1.06 (1.02–1.09)	0.001	1.03 (0.99–1.07)	0.179	1.01 (0.97–1.05)	0.590	1.01 (0.97–1.05)	0.540
**BMI**	0.99 (0.86–1.12)	0.793	1.02 (0.89–1.18)	0.763	0.99 (0.86–1.15)	0.928	1.00 (0.86–1.16)	0.950
**Body composition category**								
Sarcopenia without obesity	2.01 (0.97–4.26)	0.063	2.01 (0.95–4.33)	0.069	2.09 (0.95–4.69)	0.069	2.08 (0.95–4.69)	0.072
Obesity without sarcopenia	0.82 (0.25–2.55)	0.741	0.81 (0.25–2.55)	0.727	1.00 (0.29–3.29)	1.000	1.00 (0.29–3.30)	1.000
Sarcopenic obesity	6.97 (2.04–24.33)	0.002	6.52 (1.88–23.13)	0.003	6.86 (1.85–26.35)	0.004	7.03 (1.88–27.32)	0.004
**T-score**								
Spine			0.82 (0.60–1.11)	0.203	0.834 (0.61–1.14)	0.260	0.84 (0.61–1.15)	0.277
Hip			0.71 (0.47–1.04)	0.084	0.77 (0.50–1.15)	0.203	0.76 (0.50–1.15)	0.206
**Lab**								
Calcium					0.36 (0.18–0.66)	0.003	0.36 (0.36–0.67)	0.004
Phosphorus					0.72 (0.41–1.25)	0.249	0.72 (0.41–1.25)	0.238
Vitamin D					0.98 (0.94–1.02)	0.302	0.98 (0.93–1.02)	0.270
PTH					0.96 (0.91–1.00)	0.041	0.96 (0.91–1.00)	0.046
**Comorbidity**								
Hypertension							0.84 (0.41–1.70)	0.626
Dyslipidemia							1.04 (0.41–2.42)	0.931
Diabetes mellitus							1.01 (0.42–2.30)	0.980
Cancer							1.06 (0.29–3.61)	0.923

## Discussion

In this cross-sectional study, we divided patients into four subgroups according to their body composition to evaluate the risk of acute VOCF. Patients with sarcopenia and obesity simultaneously (sarcopenic obesity) had a significantly higher fracture rate suggesting that both weight control and muscle gain may give a protective effect to the bone health, especially in vulnerable patients. Also, this result was prominent especially for the female patients. In addition, obesity may intensify sarcopenia by acting in concert with it, resulting in physical impairment, metabolic disorders, and mortality.

Previously, these important concepts have been broadly used in various studies, and their utilization is gradually increasing. Furthermore, several studies have already reported analyzed the association between body composition and VOCF and have not reached a conclusion yet. For instance, Hida et al. found a higher prevalence of sarcopenia and lower leg muscle mass among patients with acute VOCF compared to those without acute VOCF (22). On the other hand, Anand et al. concluded that sarcopenia was not an independent risk factor for fresh vertebral fragility fractures in the elderly (23). Based on these results, we concluded that sarcopenic obesity could be an independent factor for predicting vulnerability to acute VOCF.

We found some mechanisms and supporting reasons for the findings of the present study. The back muscles that protect the spine around us are huge, and when standing up, they have a significant impact on our stability, various exercises, and daily lives. Therefore, this reduction in muscle mass not only weakens the muscle strength but also contributes to body instability. Song et al. reported that lumbar disc degeneration was correlated with multifidus degeneration (24). According to Fried et al., reduced amount of muscle mass played an important etiologic role in the frailty process of elderly subjects, being a key player in its latent phase and illustrating certain aspects of the frailty status itself (25). Interestingly, in our research, more significant results were derived from sarcopenic obesity than from sarcopenia without obesity; in particular, the results were more meaningful in the female group. Another important implication is how much weight is applied to the fractured vertebrae. Since a person with sarcopenic obesity usually has a larger force on the vertebral body during injury, it could be a critical factor for the fracture rate.

In this study, the fracture rates among four body composition groups were not significantly different in males. It can be considered in conjunction with a higher BMD in males. In many previous studies, BMD has been proven numerically or biomechanically to be positively associated with osteoporotic fractures. Ehsanbakhsh et al. reported that vertebral fractures were observed in 43.6% of osteoporotic patients, 37.5% of osteopenic patients, and 22% of patients with a normal T-score (26). Jager et al. reported that vertebral fracture prevalence was 14% in patients with normal BMD, 21% in those with osteopenia, and 33% in those with osteoporosis (27). In addition, a relatively higher muscle mass compared to that of females may also be a contributing factor.

This study has some limitations. First, as mentioned above, the number of male patients is less than the female patients and the ratio of fracture was also lower. In the further study, more male patients would be collected and we hope to find some other meaningful result regardless of gender. Second, because of the characteristics of a cross-sectional study, it was impossible to establish a real cause-and-effect relationship. It is expected that prospective research will be needed in the future to identify the causes and results throughout the periodic examination and analyses. Third, while the T-score of the spine was meaningful in the univariate study, it was not in the multivariate study, although we measured the BMD clearly according to the criteria, such as the exclusion of the vertebra with fractures. We think that this is because of the sclerotic change in the spinal body with aging. Also, unlike hip, abdominal fats in obese group could also influence the result of spine T-score (28). Finally, although the definitions of sarcopenia are various and are composed of muscle strength or function as well as muscle mass index, we used only body composition of the definition. It would be better to investigate other criteria together, but due to the limitation of the retrospective study, there was a limitation of data collection. Also, in the definition of obesity, BMI is more than 30 worldwide, but Asian countries including Korea, use 25 as a cut-off value due to differences in race and lifestyle. In order to meet the global standard, we would like to overcome those limitations above in the future study.

## Conclusion

Sarcopenic obesity can be one of the expectation factors of the acute VOCF. It could be an essential criterion for the prevention of fracture, thus finally could also be used as an fracture fragility indicator.

## Data Availability Statement

The raw data supporting the conclusions of this article will be made available by the authors, without undue reservation.

## Ethics Statement

The studies involving human participants were reviewed and approved by Institutional Review Board Approval (No. 2021-02-019). Written informed consent for participation was not required for this study in accordance with the national legislation and the institutional requirements.

## Author Contributions

YL: manuscript writing and data curation. H-JL: conceptualization, investigation, manuscript review, and editing. SY: data curation and methodology. JS and K-CP: data curation. S-yL: project administration and funding acquisition. SL: project administration, funding acquisition, and formal analysis. All authors have read and approved the manuscript.

## Funding

This work was supported by the National Research Foundation of Korea (NRF) grant funded by the Korean government (MSIT) (Nos. 2019R1G1A1095095, 2019R1C1C1004017, and 2019R1A2C4070492) and by Korea Health Technology R&D Project through the Korea Health Industry Development Institute, funded by the Ministry of Health and Welfare, Republic of Korea (Grant Number HI16C1559).

## Conflict of Interest

The authors declare that the research was conducted in the absence of any commercial or financial relationships that could be construed as a potential conflict of interest.

## Publisher's Note

All claims expressed in this article are solely those of the authors and do not necessarily represent those of their affiliated organizations, or those of the publisher, the editors and the reviewers. Any product that may be evaluated in this article, or claim that may be made by its manufacturer, is not guaranteed or endorsed by the publisher.
