# Supplementary material for: Association Between Overweight Sarcopenic Population and Acute Vertebral Osteoporotic Compression Fractures in Females: Retrospective, Cross-Sectional Study
Source: Front Med (Lausanne). 2021 Dec 3;8:790135. doi: 10.3389/fmed.2021.790135 (PMC8678084; doi:10.3389/fmed.2021.790135)
Supplement: Supplementary file 1 [file Table_1.DOCX]

Supplementary Material

|  | **Model 1** | | **Model 2** | | **Model 3** | | **Model 4** | |
| --- | --- | --- | --- | --- | --- | --- | --- | --- |
|  | AIC = 367.32 | | AIC = 364.8 | | AIC = 351.89 | | AIC = 359.23 | |
| Variable | OR (95% CI) | *p* value | OR (95% CI) | *p* value | OR (95% CI) | *p* value | OR (95% CI) | *p* value |
| **Diagnosed age** | 1.05 (1.02-1.08) | < 0.001 | 1.03 (1.00-1.07) | 0.041 | 1.02 (0.98-1.05) | 0.34 | 1.02 (0.98-1.05) | 0.335 |
| **BMI** | 1.01 (0.89-1.14) | 0.904 | 1.03 (0.91-1.17) | 0.616 | 1.03 (0.90-1.18) | 0.671 | 1.03 (0.90-1.18) | 0.67 |
| **Body shape** |  |  |  |  |  |  |  |  |
| Sarcopenia without obesity | 1.73 (0.89-3.44) | 0.109 | 1.69 (0.86-3.38) | 0.132 | 1.50 (0.74-3.09) | 0.268 | 1.49 (0.73-3.07) | 0.28 |
| Obesity without sarcopenia | 0.77 (0.27-2.13) | 0.621 | 0.78 (0.27-2.18) | 0.643 | 0.88 (0.30-2.53) | 0.818 | 0.88 (0.29-2.54) | 0.819 |
| Sarcopenic obesity | 4.71 (1.64-13.59) | 0.004 | 5.01 (1.72-14.71) | 0.003 | 4.66 (1.53-14.37) | 0.007 | 4.53 (1.48-14.01) | 0.008 |
| **T-score** |  |  |  |  |  |  |  |  |
| spine |  |  | 0.85 (0.65-1.10) | 0.22 | 0.86 (0.65-1.12) | 0.265 | 0.85 (0.64-1.11) | 0.227 |
| hip |  |  | 0.82 (0.60-1.11) | 0.205 | 0.82 (0.59-1.12) | 0.207 | 0.83 (0.60-1.13) | 0.239 |
| **Lab** |  |  |  |  |  |  |  |  |
| Calcium |  |  |  |  | 0.46 (0.26-0.75) | 0.004 | 0.47 (0.27-0.76) | 0.005 |
| Phosphorus |  |  |  |  | 0.72 (0.45-1.17) | 0.188 | 0.74 (0.45-1.20) | 0.225 |
| Vitamin D |  |  |  |  | 0.99 (0.95-1.02) | 0.477 | 0.99 (0.95-1.03) | 0.539 |
| PTH |  |  |  |  | 0.95 (0.91-0.99) | 0.015 | 0.95 (0.91-0.99) | 0.022 |
| **Comorbidity** |  |  |  |  |  |  |  |  |
| Hypertension |  |  |  |  |  |  | 1.08 (0.59-1.95) | 0.81 |
| Dyslipidemia |  |  |  |  |  |  | 0.87 (0.38-1.88) | 0.741 |
| Diabetes mellitus |  |  |  |  |  |  | 1.19 (0.58-2.39) | 0.622 |
| Cancer |  |  |  |  |  |  | 1.25 (0.42-3.40) | 0.679 |
| OR, odds ratio; CI, confidence interval | |  |  |  |  |  |  |  |

**Supplementary Table 1.** Multivariate logistic regression analysis of factors associated with acute VOCF for total group

|  | **Model 1** | | **Model 2** | | **Model 3** | | **Model 4** | |
| --- | --- | --- | --- | --- | --- | --- | --- | --- |
|  | AIC = 83.048 | | AIC = 86.687 | | AIC = 84.993 | | AIC = 89.901 | |
| Variable | OR (95% CI) | *p* value | OR (95% CI) | *p* value | OR (95% CI) | *p* value | OR (95% CI) | *p* value |
| **Diagnosed age** | 1.05 (0.98-1.13) | 0.209 | 1.05 (0.97-1.13) | 0.212 | 1.02 (0.93-1.12) | 0.64 | 1.02 (0.93-1.13) | 0.658 |
| **BMI** | 1.07 (0.78-1.48) | 0.683 | 1.010 (0.78-1.54) | 0.59 | 1.24 (0.84-1.86) | 0.292 | 1.12 (0.73-1.78) | 0.605 |
| **Body shape** |  |  |  |  |  |  |  |  |
| Sarcopenia without obesity | 1.14 (0.21-7.01) | 0.878 | 1.15 (0.20-7.45) | 0.874 | 0.56 (0.06-4.67) | 0.584 | 0.42 (0.04-4.08) | 0.452 |
| Obesity without sarcopenia | 0.82 (0.06-9.42) | 0.878 | 0.72 (0.05-8.63) | 0.802 | 0.81 (0.05-10.78) | 0.872 | 1.54 (0.08-27.09) | 0.765 |
| Sarcopenic obesity | 2.16 (0.22-20.84) | 0.498 | 2.05 (0.21-19.88) | 0.527 | 2.50 (0.21-30.93) | 0.46 | 4.06 (0.25-74.70) | 0.325 |
| **T-score** |  |  |  |  |  |  |  |  |
| spine |  |  | 0.834(0.44-1.50) | 0.567 | 0.72 (0.37-1.35) | 0.322 | 0.67 (0.32-1.28) | 0.24 |
| hip |  |  | 1.16 (0.60-2.22) | 0.654 | 1.30 (0.65-2.62) | 0.45 | 1.47 (0.68-3.26) | 0.325 |
| **Lab** |  |  |  |  |  |  |  |  |
| Calcium |  |  |  |  | 0.31 (0.07-1.23) | 0.1 | 0.19 (0.03-0.91) | 0.05 |
| Phosphorus |  |  |  |  | 0.57 (0.15-1.98) | 0.382 | 0.39 (0.08-1.72) | 0.223 |
| Vitamin D |  |  |  |  | 1.04 (0.94-1.16) | 0.417 | 1.04 (0.93-1.18) | 0.48 |
| PTH |  |  |  |  | 0.86 (0.75-0.96) | 0.012 | 0.84 (0.72-0.95) | 0.01 |
| **Comorbidity** |  |  |  |  |  |  |  |  |
| Hypertension |  |  |  |  |  |  | 2.64 (0.52-17.74) | 0.268 |
| Dyslipidemia |  |  |  |  |  |  | 0.49 (0.02-4.79) | 0.596 |
| Diabetes mellitus |  |  |  |  |  |  | 2.28 (0.32-17.09) | 0.405 |
| Cancer |  |  |  |  |  |  | 0.36 (0.02-4.79) | 0.473 |
| OR, odds ratio; CI, confidence interval | |  |  |  |  |  |  |  |

**Supplementary Table 2.** Multivariate logistic regression analysis of factors associated with acute VOCF for male group
